# P-610. Estimating Risk of Guillain-Barré Syndrome in US Medicare-Enrolled Older Adults Following Medically Attended Respiratory Syncytial Virus Disease: A Self-Controlled Case Series Analysis

**DOI:** 10.1093/ofid/ofaf695.823

**Published:** 2026-01-11

**Authors:** Jennifer Judy, Caihua Liang, Erica Chilson, Scott P Kelly, Qing Liu, Jason D Maynard, Heidi De Souza, Bradford D Gessner, Elizabeth Begier

**Affiliations:** Pfizer, New York, New York; Pfizer Inc, New York, NY; Pfizer, New York, New York; Pfizer, New York, New York; Pfizer Inc., Collegeville, Pennsylvania; ADVI Health, Washington DC, District of Columbia; ADVI Health LLC, Washington, District of Columbia; EpiVac Consulting, Anchorage, Alaska; Pfizer Vaccines, Dublin, Dublin, Ireland

## Abstract

**Background:**

Guillain-Barré syndrome (GBS) may be preceded by viral respiratory infections (e.g., influenza and SARS-CoV-2) and is considered a rare complication of such infections. However, the association between GBS and RSV infection has not been described. We performed a self-controlled case series analysis to estimate GBS risk associated with medically attended RSV disease among older adults.

**Figure:**
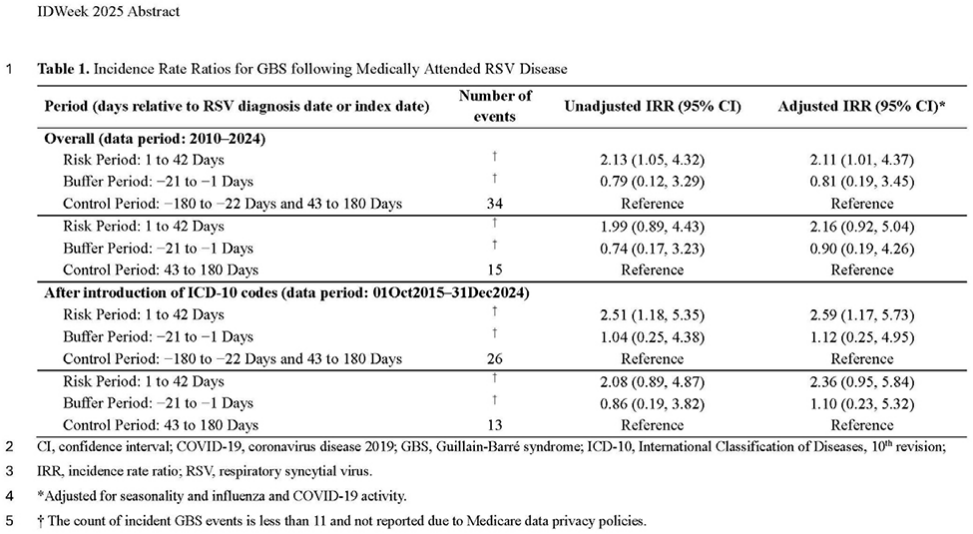

**Methods:**

Adults aged ≥65 years who had both RSV infection and incident GBS were identified from US Medicare Part A, B, and C claims data (2010–2024). RSV disease was identified via outpatient or inpatient visits’ ICD codes (J12.1, J20.5, J21.0, B97.4, 079.6, 480.1, 466.11, or 466.1 as primary or secondary diagnosis). GBS events had a primary ICD code of G61.0 or 357.0 in an inpatient setting or any setting if hospitalized in following 7 days. Patients with GBS ICD codes during year preceding RSV disease were excluded. The risk period was 1–42 days after RSV disease, with a predefined buffer period of 21 days preceding RSV index date (earliest date of outpatient visit or inpatient admission). The control period encompassed the remaining 1-year observation period (−180 to −22 days and 43–180 days). Conditional Poisson regression was used to estimate RSV-related GBS incidence rate ratios (IRRs) with adjustment for time-varying covariates (e.g., seasonality and influenza and COVID-19 activity). We conducted sensitivity analyses restricting to after ICD-10 introduction and using a post-infection only control period. Medicare data policies prohibit cell case counts < 11 from being displayed.

**Results:**

We identified 452,471 eligible patients with medically attended RSV disease. Of these, < 11 incident GBS cases were observed in the risk period and 34 cases in the control period (Table). The adjusted IRR for RSV-associated GBS was 2.11 (95% CI: 1.01–4.37). The IRR remained consistent when using a control period of 43–180 days (2.16 [0.92–5.04]) and increased when restricted to the period after ICD-10 code introduction (2.59 [1.17–5.73]).

**Conclusion:**

GBS risk is increased after medically attended RSV disease compared to control periods not adjacent to RSV disease. RSV should be recognized as one of the viral respiratory pathogens that can lead to the rare complication of GBS.

**Disclosures:**

Jennifer Judy, MS, PhD, Pfizer: I am an employee|Pfizer: Stocks/Bonds (Public Company) Caihua Liang, MD, PhD, Pfizer: I am an employee.|Pfizer: Stocks/Bonds (Public Company)|Pfizer: Stocks/Bonds (Public Company) Erica Chilson, PharmD, Pfizer Inc: Stocks/Bonds (Public Company) Scott P. Kelly, PhD, Pfizer: Stocks/Bonds (Public Company) Qing Liu, M.S., Pfizer Inc.: I am Pfizer employee and hold Pfizer stocks|Pfizer Inc.: Stocks/Bonds (Public Company) Heidi De Souza, MPH, ADVI Health LLC: Employee; may hold company shares and/or stocks. Bradford D. Gessner, MD, MPH, Pfizer: Stocks/Bonds (Public Company) Elizabeth Begier, MD, M.P.H., Pfizer: I am an employee.|Pfizer: Stocks/Bonds (Public Company)

